# Methylome-wide association study of different responses to risperidone in schizophrenia

**DOI:** 10.3389/fphar.2022.1078464

**Published:** 2022-12-22

**Authors:** Huihui Du, Jingsong Ma, Wei Zhou, Mo Li, Cong Huai, Lu Shen, Hao Wu, Xianglong Zhao, Na Zhang, Songyin Gao, Qi Wang, Lin He, Xuming Wu, Shengying Qin, Mingzhe Zhao

**Affiliations:** ^1^ Bio-X Institutes, Key Laboratory for the Genetics of Developmental and Neuropsychiatric Disorders Ministry of Education, Shanghai Jiao Tong University, Shanghai, China; ^2^ School o f Engineering, Westlake University, Hangzhou, Zhejiang, China; ^3^ Institute of Advanced Technology, Westlake Institute for Advanced Study, Hangzhou, Zhejiang, China; ^4^ Zhumadian Psychiatric Hospital, Zhumadian, China; ^5^ Hebei Mental Health Center, Hebei Sixth People’s Hospital, Baoding, China; ^6^ Nantong Fourth People’s Hospital, Nantong, China; ^7^ Affiliated Mental Health Center and Hangzhou Seventh People’s Hospital, Zhejiang University School of Medicine, Hangzhou, China

**Keywords:** pharmacoepigenomics, antipsychotic response, MWAS, DMP, DMR

## Abstract

**Background:** Accumulating evidence shows that DNA methylation plays a role in antipsychotic response. However, the mechanisms by which DNA methylation changes are associated with antipsychotic responses remain largely unknown.

**Methods:** We performed a methylome-wide association study (MWAS) to evaluate the association between DNA methylation and the response to risperidone in schizophrenia. Genomic DNA methylation patterns were assessed using the Agilent Human DNA Methylation Microarray.

**Results:** We identified numerous differentially methylated positions (DMPs) and regions (DMRs) associated with antipsychotic response. *CYP46A1*, *SPATS2*, and *ATP6V1E1* had the most significant DMPs, with *p* values of 2.50 × 10^–6^, 3.53 × 10^–6^, and 5.71 × 10^–6^, respectively. The top-ranked DMR was located on chromosome 7, corresponding to the *PTPRN2* gene with a Šidák-corrected *p*-value of 9.04 × 10^–13^. Additionally, a significant enrichment of synaptic function and neurotransmitters was found in the differentially methylated genes after gene ontology and pathway analysis.

**Conclusion:** The identified DMP- and DMR-overlapping genes associated with antipsychotic response are related to synaptic function and neurotransmitters. These findings may improve understanding of the mechanisms underlying antipsychotic response and guide the choice of antipsychotic in schizophrenia.

## Introduction

Schizophrenia (OMIM 181500) is a severe psychiatric disorder defined by the presence of symptoms including hallucinations, delusions, disorganized speech and behavior, diminished emotional expression, social withdrawal and cognitive impairment, which has a lifetime risk of approximately .7% (McGrath et al., 2008) and a prevalence of about 1% (Greenwood et al., 2019). The onset of schizophrenia is referred to as the prodromal phase and comprises a decline in cognitive and social functions, which generally begins in the early adolescent years and precedes the onset of psychotic symptoms by more than ten years (Kahn and Keefe, 2013). Schizophrenia is highly heritable (Cardno and Gottesman, 2000) and many genetic variants involved in the risk architecture of schizophrenia have been identified across diverse populations (Weinberger, 2019). For the last 70 years, antipsychotics have been the only effective treatment for the disorder (Lisoway et al., 2021), however, only 50% of schizophrenia patients show favorable symptom improvement (Lohoff and Ferraro, 2010). Furthermore, existing therapies largely relieve primarily positive symptoms (hallucinations and delusions), and the response to existing antipsychotic medications is highly variable (Lehman et al., 2004). The lack of progress in therapeutic development is a consequence of the limited understanding of the molecular etiology of antipsychotic response (Yu H. et al., 2018).

Due to the possible role of genetic factors in antipsychotic treatment effectiveness, pharmacogenomics has become a significant consideration for improving treatment outcomes in schizophrenia (Soria-Chacartegui et al., 2021). Recently, a genome-wide association study (GWAS) and a whole-exome sequencing (WES) study have revealed a series of genetic variants associated with antipsychotic response (Wang et al., 2018; Yu H. et al., 2018). Despite advances in the pharmacogenomics of schizophrenia, the biological functions of the majority of identified variants are unclear. Moreover, none of the variants identified thus far are sufficiently informative to predict antipsychotic response (Lisoway et al., 2021). In light of this, pharmacoepigenomics is emerging as an important player in the search for the biological architecture of the response to antipsychotic treatment (Lisoway et al., 2021).

The investigation of epigenetic alterations and their relationship to antipsychotic response may be useful in the discovery of pertinent, non-invasive biomarkers to advance personalized psychiatric medicine. In humans, as one of the major types of epigenetic modifications, and perhaps the most well-characterized, DNA methylation refers primarily to a biological process that adds a methyl group to the cytosines of CpG dinucleotides (Greenberg and Bourc’his, 2019). Studies in antipsychotic treatment have focused on investigating the associations between antipsychotic response and DNA methylation of the promoter regions of candidate genes, such as *SLC6A4* (Abdolmaleky et al., 2014), *HTR1A* (Tang et al., 2014), and *BDNF* (Dong et al., 2016). However, this strategy cannot provide a comprehensive landscape of how epigenetics affects the antipsychotic response. As genome-wide methylation scanning technology evolves, methylome-wide association studies (MWASs) have been adopted across different fields, and has successfully identified promising methylation biomarkers associated with drugs. For example, Murata.et al. used the HumanMethylation450 BeadChip (Illumina) to conduct a genome-wide assessment of human neuroblastoma cells that had received blonanserin at two different dosages (Murata et al., 2014). The authors identified 1657 hyper- and 1104 hypomethylated CpG sites in both dose groups compared to controls. Furthermore, these CpG sites were enriched outside of gene promoters, 3′-, and intergenic regions. However, there have been few studies on the association between DNA methylation and antipsychotic response at the genome-wide level. MWAS may provide additional insightful information for the traditional pharmacogenomic research.

The objective of the current study was to scan genome-wide DNA methylation data to identify differentially methylation biomarkers of antipsychotic response in blood. To analyze the association between DNA methylation and antipsychotic response, we applied both genome-wide position-based and region-based approaches (Li et al., 2021). Gene set analysis was carried out to shed light on coherent biological pathways and processes.

## Materials and methods

### Study samples

In total, 86 schizophrenic patients were recruited from our clinical networks in Shanghai. Diagnosis and blood sample collection were performed by skilled psychiatrists. Patients with schizophrenia were enrolled in the study if they satisfied the following requirements: 1) they satisfied the criteria for schizophrenia in the Structured Clinical Interview of the Diagnosis and Statistical Manual of Mental Disorder Fourth Edition (DSM-IV); 2) they ranged in age from 18 to 50; 3) physically fit with all laboratory results falling within acceptable ranges; and 4) took pills orally. Before participating in this study, all patients were required to designate a legal guardian to give written informed permission and assist patients in making decisions. The Bio-X Institute’s Ethical Committee at Shanghai Jiao Tong University gave its approval for this project. All of the participants were Han Chinese.

### Phenotypic assessment

The Positive and Negative Syndrome Scale (PANSS) was used to evaluate clinical symptoms. The percentage change in PANSS to antipsychotic medication was used to assess treatment response. During an 8-week risperidone treatment, medication efficacy was assessed four times (baseline, week two, week four and week eight) using the difference in PANSS scores from baseline. The following equation was used to determine each participant’s PANSS reduction rate at week eight (Obermeier et al., 2010; Yu H. et al., 2018).

$$PANSS\;percentage\;change=\frac{PANSS\;baseline\;scores-PANSS\;week\;eight\;scores\;}{PANSS\;baseline\;scores-30}\times 100\%$$

### DNA methylation detection

Following the instructions on the QIAamp DNA Blood Kits (Qiagen, United States of America), genomic DNA was obtained from whole blood samples. The methylation status was evaluated using the Agilent Human DNA Methylation Microarray (1 × 244 k; Agilent Technologies, Santa Clara, CA) according to the manufacturer’s recommendations. Briefly, 4 μg of DNA was broken up into 200–1000 bps. Eighty percent of the shared DNA was then immunoprecipitated using anti-5-methylcytidine monoclonal antibody, with the remaining 20% serving as a control. The DNA fragments with two sections were purified, tagged, and then hybridized onto microarray slides. An Agilent SureScan Microarray Scanner G2505C was used to create images of slides, which were then converted to digital features using Agilent Feature Extraction program (version 10.7.1.1). The Agilent Genomic Workbench CH3 application (version 7.0) was used to examine the feature data for quality control. The methylation status of all probes was determined by the Bayesian tool for methylation analysis (BATMAN) (Down et al., 2008). In the BATMAN analysis, the methylation status was categorized into three levels: “1” (high, DNA methylation >60%), “0” (moderate, DNA methylation 40–60%) and “-1” (low, DNA methylation <40%) (Down et al., 2008). Probes with poor quality (outlier flag = 1) in >10% of the subjects, associated with cellular specificity (Jaffe and Irizarry, 2014), located on sex chromosomes and BATMAN values for probes that concentrated to one level in >95% of the subjects were excluded from the remaining analyses (Huai et al., 2019; Shen et al., 2021).

### Statistical analysis

To identify the association between differentially methylated positions (DMPs) and PANSS percentage change values, we used linear regression adjusting for age (Yu H. et al., 2018). The identified DMPs were annotated by Table Browser implemented in the UCSC genome browser (http://genome.ucsc.edu/cgi-bin/hgTables) (Shen et al., 2021). Differentially methylated regions (DMRs) were determined using *comb-p* (Pedersen et al., 2012) with a distance of 500 bp and a seeded *p*-value of .01. The DMR analysis was carried out for all probes, and DMRs were considered significant and reported if they were three or more and with Šidák-corrected *p* less than .05. Then we used Bedtools to intersect the GTF files and determine the genomic annotation of the identified DMRs. GTF files were downloaded from GENCODE (https://www.gencodegenes.org/human/release_40lift37.html). The Human Protein Atlas database (https://www.proteinatlas.org) was used to analyze the expression patterns of DMP- and DMR-overlapping genes in the human brain (Yu et al., 2015).

### Gene ontology enrichment and network analysis

To further understand the function of the overlapping genes mentioned above, the STRING v11.5 database was used to perform gene ontology (GO) analysis, pathway analysis and protein‒protein interaction (PPI) network analysis (Szklarczyk et al., 2019). Go and pathway terms were restricted to those that satisfied an adjusted *p*-value <.05 (false discovery rate, FDR) threshold. The detailed protein‒protein interaction network graphs were drawn using Cytoscape 3.9.1 (Shannon et al., 2003).

### Gene set analysis

GeneAnalytics (https://geneanalytics.genecards.org) was used to perform gene set analysis on a combined dataset of DMPs and DMRs (Ben-Ari Fuchs et al., 2016). The rationale behind combining DMPs and DMRs into a single group for gene set analysis was to address the problem that not all probes may create DMRs. DMPs and DMRs were investigated jointly to give those regions of the genome a chance to contribute to gene set analysis.

## Results

### Identification of differentially methylated positions

**FIGURE 1:**
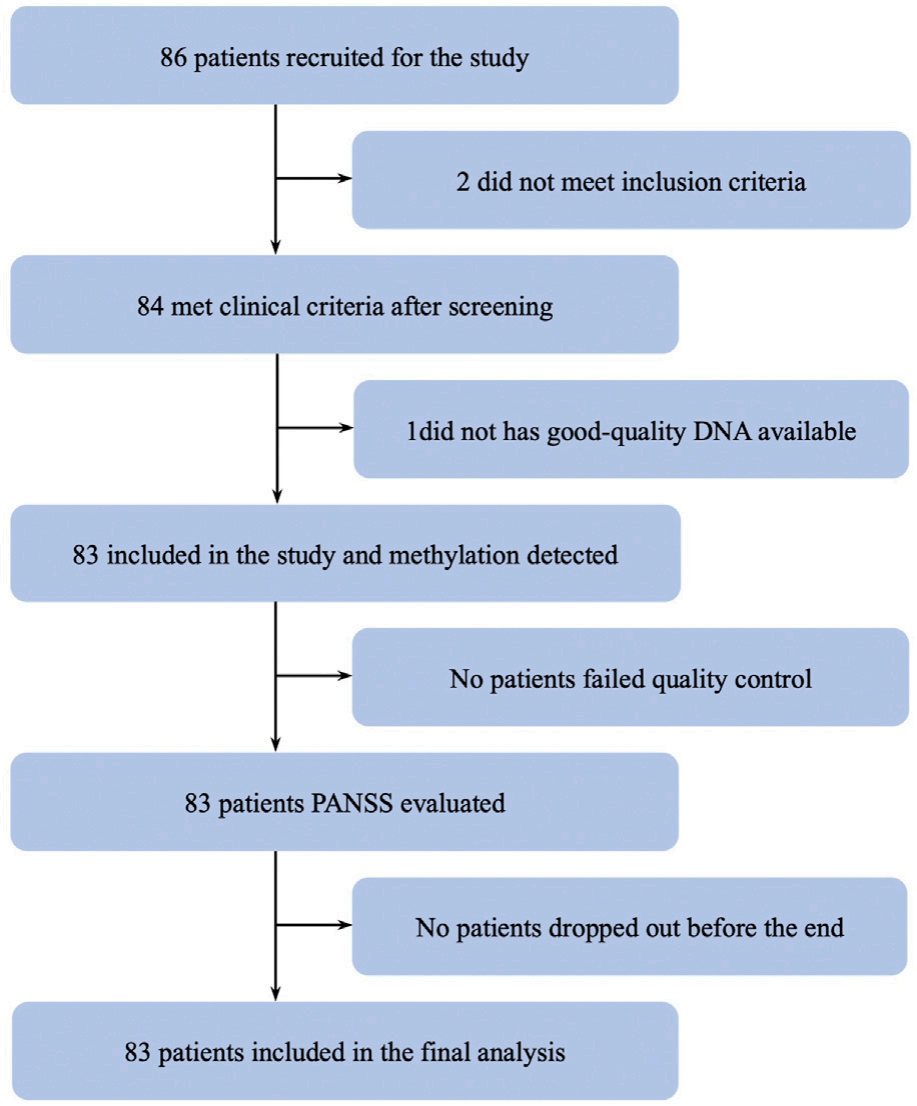
Trial profile for samples.

A total of 83 schizophrenic patients (37.7 ± 11.2 years; 45 males and 38 females) met the inclusion criteria (Figure 1). The DNA methylation status of the peripheral blood cells was detected using the Agilent Human 1 × 244 k DNA Methylation Microarray. After quality control, a total of 229,786 methylation probes were retained to perform whole-genome methylation analysis.

**FIGURE 2:**
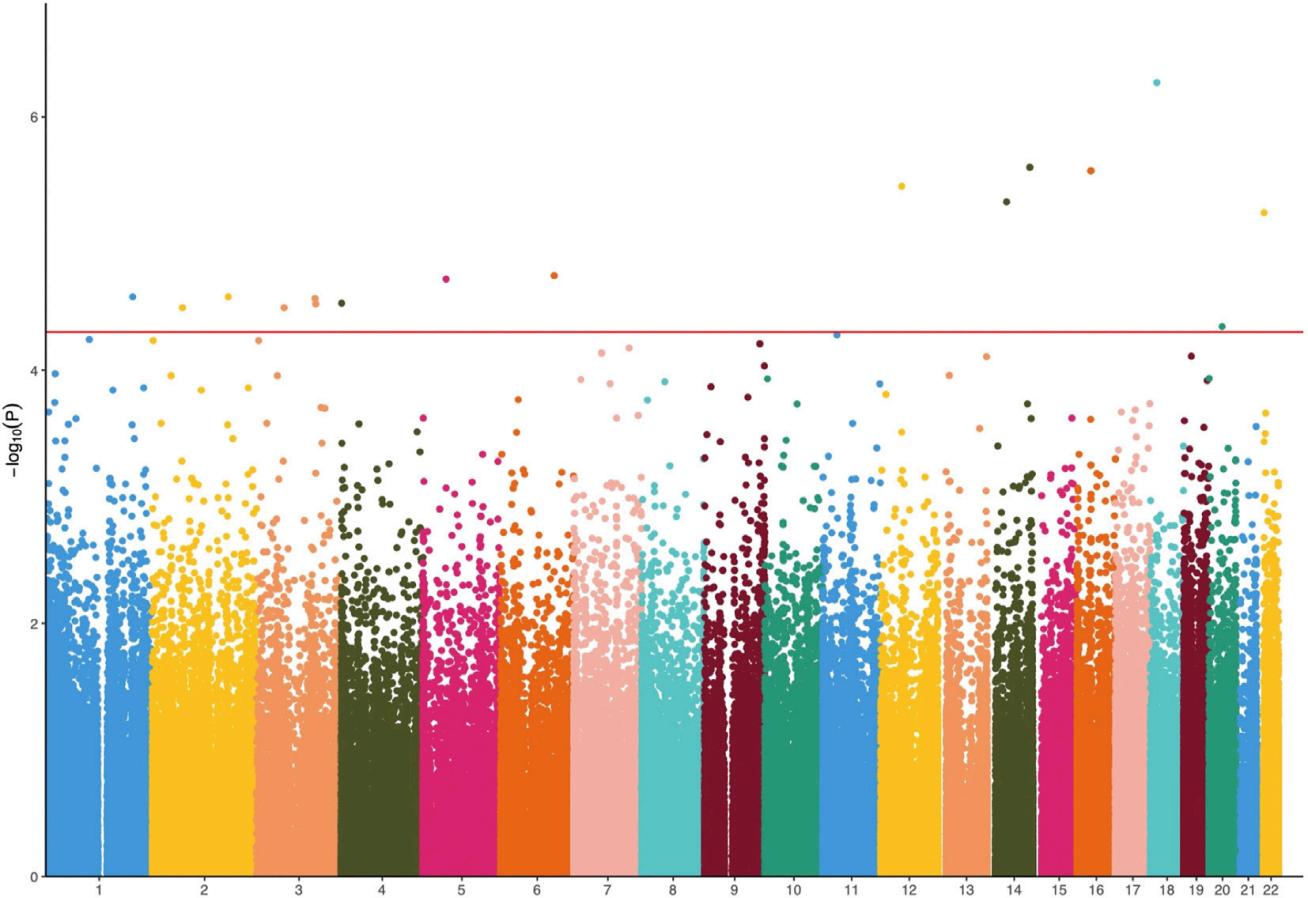
Manhattan plots of *p* values against their respective chromosomal positions for methylome-wide association study. The red line represents the genome-wide significance level (*p* < 5 × 10^–5^).

The results of linear regression analysis demonstrated that 17 DMPs exhibited strong association with PANSS percentage change values (*p* < 5 × 10^–5^; Figure 2 and Table 1). These DMPs were mapped to 11 unique genes. The most significant DMPs were annotated to genes *CYP46A1*, *SPATS2*, and *ATP6V1E1* with *p* values of 2.50 × 10^–6^, 3.53 × 10^–6^, and 5.71 × 10^–6^, respectively. There were two other DMPs (probe 176,224: Chr16:32096066–32096110 and probe 176,445: Chr16:33039318–33039362) located in the chromosome 16p11.2 region which has been consistently highlighted in genetic studies of schizophrenia and antipsychotic response (McCarthy et al., 2009; Lit et al., 2012; Chang et al., 2017; Marshall et al., 2017) (Table 1). More importantly, five genes (*NME9*, *CPLX1*, *CYP46A1*, *PTPRN2*, and *S1PR5*) were highly expressed in brain tissue (Supplementary Figure S1).

**TABLE 1:** Differentially methylated positions associated with antipsychotic response.

Probe id	Chr	Position	Beta	p-Value	Gene annotation
Probe199512	18	15198183–15198229	−5.495	5.35 × 10–7	NA
Probe159068	14	99712034–99712078	−6.46	2.50 × 10–6	CYP46A1
Probe176224	16	32096066–32096110	−4.925	2.66 × 10–6	NA
Probe176445	16	33039318–33039362	−4.925	2.66 × 10–6	NA
Probe141649	12	49391405–49391449	−6.479	3.53 × 10–6	SPATS2
Probe155762	14	45366839–45366883	−5.001	4.68 × 10–6	NA
Probe234347	22	17600992–17601036	4.571	5.71 × 10–6	ATP6V1E1
Probe77498	6	125421078–125421122	−4.856	1.79 × 10–5	ENSG00000226409
Probe63163	5	53815136–53815180	−5.375	1.91 × 10–5	LINC02105
Probe21003	1	202927483–202927527	−4.358	2.63 × 10–5	S1PR5
Probe36053	2	176969323–176969367	−4.358	2.63 × 10–5	NA
Probe47200	3	136471624–136471668	−4.272	2.71 × 10–5	STAG1
Probe50932	4	789,387–789431	−4.807	2.96 × 10–5	CPLX1
Probe47358	3	138312523–138312573	4.409	3.00 × 10–5	NME9
Probe29675	2	70142804–20142848	−4.63	3.21 × 10–5	NA
Probe44725	3	64430588–64430632	−4.63	3.21 × 10–5	PRICKLE2
Probe225810	20	30539585–30539629	−4.226	4.52 × 10–5	ZNF285DP

Chr chromosome.

### Identification of differentially methylated regions

The DMR analysis, which enabled us to identify regions of the genome with ≥3 probes, revealed a total of 21 DMRs significantly associated with antipsychotic response (Šidák-corrected *p*-value <.05; Table 2), overlapping with 21 unique genes. The two most significant DMRs were located on Chr7:157524168–157526728 (*p*_Šidák_ = 9.04 × 10^–13^) and Chr 9: 140735579–140737213 (*p*_Šidák_ = 2.69 × 10^–11^), corresponding to the *PTPRN2* gene and *EHMT1*gene, respectively. Of the 21 genes, six genes (*ATP9B*, *CFAP46*, *GRIN1*, *PLEKHG4B*, *PTPRN2*, and *TUBB3*) were highly expressed in brain tissue (Supplementary Figure S2).

**TABLE 2:** Differentially methylated regions associated with antipsychotic response.

Chr	Position	*p*-Value	*p*_Šidák_	Gene annotation
7	157524168–157526728	2.01 × 10–16	9.04 × 10–13	*PTPRN2*
9	140735579–140737213	4.25 × 10–15	2.69 × 10–11	*EHMT1*
17	205,530–206388	5.12 × 10–14	6.21 × 10–10	*AC129507.2*
17	205,530–206388	5.12 × 10–14	6.21 × 10–10	*AC129507.3*
17	205,530–206388	5.12 × 10–14	6.21 × 10–10	*RPH3AL*
7	1155973–1157407	1.03 × 10–12	7.51 × 10–9	*C7orf50*
5	164,915–166649	1.34 × 10–12	8.07 × 10–9	*PLEKHG4B*
10	367,595–369374	3.91 × 10–12	2.29 × 10–8	*DIP2C*
9	140045085–140045825	4.40 × 10–12	6.19 × 10–8	*GRIN1*
8	1509976–1511253	2.77 × 10–10	2.26 × 10–6	*DLGAP2*
4	187292480–187293559	8.11 × 10–10	7.83 × 10–6	*F11-AS1*
2	306,787–308585	8.68 × 10–9	5.03 × 10–5	*AC079779.3*
5	1339440–1340060	3.82 × 10–9	6.41 × 10–5	*CLPTM1L*
4	752,689–753634	8.66 × 10–9	9.55 × 10–5	*PCGF3*
10	134731367–134731667	9.35 × 10–9	.0003245	*CFAP46*
17	3590017–3590955	9.26 × 10–8	.001028	*P2RX5-TAX1BP3*
17	3590017–3590955	9.26 × 10–8	.001028	*P2RX5*
16	90000405–90000666	1.36 × 10–7	.0054	*AC092143.1*
16	90000405–90000666	1.36 × 10–7	.0054	*TUBB3*
18	76906018–76906464	3.43 × 10–7	.007986	*ATP9B*
16	86006795–86007409	5.50 × 10–7	.009292	*AC092723.4*

*Chr* chromosome, *p*_Šidák_ *p* values adjusted by the Šidák correction method.

### GO enrichment profiling and network analysis

**FIGURE 3:**
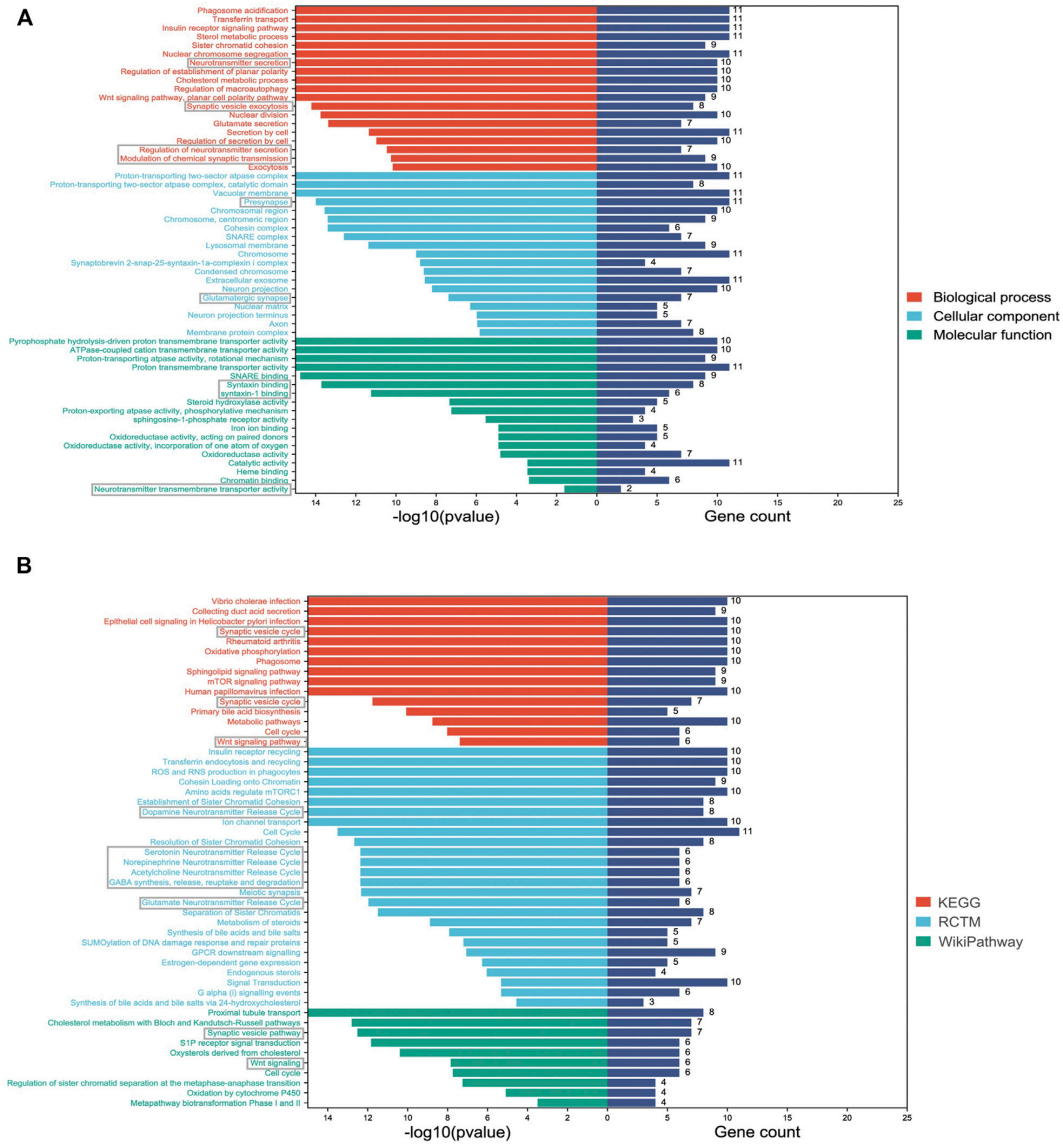
Significantly enriched GO and pathway terms of DMP-related genes. **(A)** The *Y*-axis represents the GO terms and the *X*-axis represents the *p* values and gene count of enrichment; **(B)** The *Y*-axis represents the pathway terms and the *X*-axis represents the *p* values and gene count of enrichment. Synaptic function and neurotransmitters-related terms are labeled with grey boxes.

To describe common characteristics of the DMPs, we performed GO and pathway enrichment analyses. The top 20 GO terms were listed in the categories of biological processes, cellular components and molecular functions according to the FDR values (Figure 3A). The functions of the differentially methylated genes were mainly involved in synaptic functions and neurotransmitter processes, such as synaptic vesicle exocytosis, presynapse, syntaxin binding, neurotransmitter secretion, and neurotransmitter transmembrane transporter activity. Pathway analysis was performed utilizing the Kyoto Encyclopedia of Genes and Genomes (KEGG) database, Reactome Pathway database, and WikiPathways database. The top-ranked enriched terms were involved in synapse- and neurotransmitters-related pathways, such as the synaptic vesicle pathway, dopamine neurotransmitter release cycle, and serotonin neurotransmitter release cycle (Figure 3B). Additionally, the Wnt signaling pathway, known to be crucial for neurodevelopment and nervous system regulation (Meffre et al., 2014; Yu Z. et al., 2018), was also significantly enriched (Figure 3B).

**FIGURE 4:**
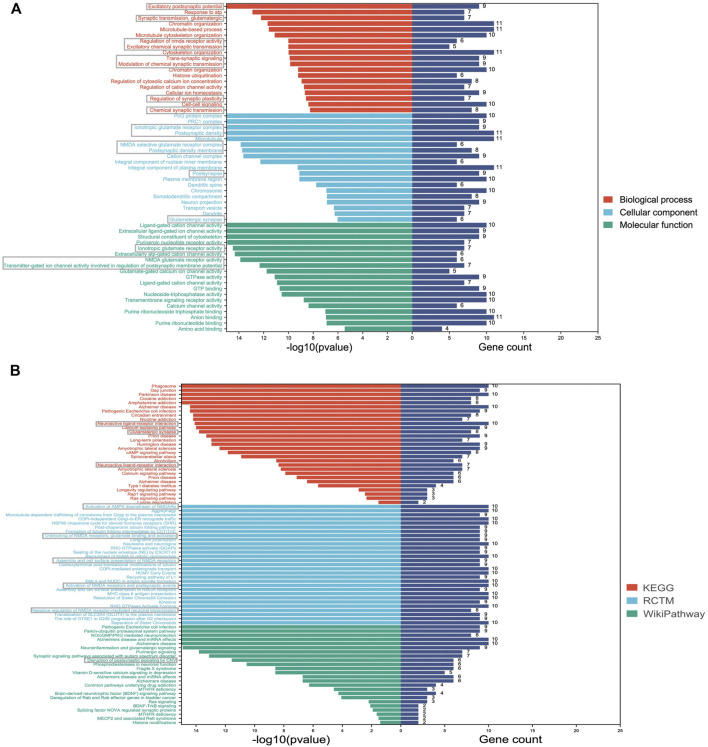
Significantly enriched GO and pathway terms of DMR-related genes. **(A)** The *Y*-axis represents the GO terms and the *X*-axis represents the *p* values and gene count of enrichment; **(B)** The *Y*-axis represents the pathway terms and the *X*-axis represents the *p* values and gene count of enrichment. Synaptic and glutamate function-related terms are labeled with grey boxes.

Consistent with the DMP results, DMR-related genes were also linked to synaptic functions (e.g., excitatory postsynaptic potential, synaptic transmission, postsynaptic density, and postsynaptic density membrane) (Figure 4A). In addition, we identified several GO terms related to glutamate functions, such as ionotropic glutamate receptor activity, NMDA glutamate receptor activity, glutamate-gated calcium ion channel activity, and NMDA selective glutamate receptor complex (Figure 4A). Consistent with the GO results, the top-ranked enriched terms from the three pathway databases were centered on glutamate related pathways, especially NMDA related pathways, such as activation of AMPK downstream of NMDARs, unblocking of NMDA receptors, glutamate binding and activation, assembly and cell surface presentation of NMDA receptors (Figure 4B).

Two PPI networks were obtained by investigating PPI *via* the STRING database using DMP- and DMR-overlapping genes and previously reported candidate genes that showed a positive association with antipsychotic response (Supplementary Figure S3) (Yu H. et al., 2018). As shown in Supplementary Figure S3a, all DMP-overlapping genes can construct a PPI network with half of the DMR-overlapping genes. Furthermore, we found a highly interconnected PPI network constructed by the DMP-overlapping genes, DMR-overlapping genes, and candidate genes. (Supplementary Figure S3b).

### Gene set analysis

Detailed outcomes for combined gene set enrichment of DMPs and DMRs can be found in Table 3. In short, the top-two enriched tissues were brain and neurons; the top-two enriched systems were nervous system and sensory organs; the top-two enriched diseases were schizophrenia and autosomal dominant non-syndromic intellectual disability; and the top-two enriched human phenotype ontologies (HPO) were abnormal hippocampal morphology and abnormal morphology of the limbic system. For the GO and pathway enrichment analyses, the top-ranked enriched terms were also concentrated on synaptic functions.

**TABLE 3:** Gene sets analysis for DMPs and DMRs.

Variable	Significant genes in MWAS	Match genes	Total genes	Score
Tissues				
Brain	*ATP6V1E1, CPLX1, CYP46A1, DIP2C, GRIN1, P2RX5, PCGF3, PRICKLE2, PTPRN2, STAG1, TUBB3*	dsl11	7803	1.72
Neurons	*CPLX1, PRICKLE2, TUBB3*	3	1667	.93
Eye	*ATP6V1E1, CPLX1, GRIN1, PRICKLE2, S1PR5, TUBB3*	6	3794	.87
Neural Tube	*ATP6V1E1, CPLX1, GRIN1, RPH3AL, STAG1, TUBB3*	7	2916	.77
Spinal Cord	*ATP6V1E1, CPLX1, GRIN1, TUBB3*	4	2967	.72
				
System				
Nervous System	*ATP6V1E1, CPLX1, CYP46A1, DIP2C, GRIN1, P2RX5, PCGF3, PRICKLE2, PTPRN2, RPH3AL, STAG1, TUBB3*	12	8478	1.81
Sensory Organs	*ATP6V1E1, CPLX1, GRIN1, PRICKLE2, S1PR5, TUBB3*	6	4350	.87
Gastrointestinal Tract	*ATP6V1E1, C7orf50, CLPTM1L, CPLX1, PRICKLE2, PTPRN2, RPH3AL*	7	6900	.65
Reproductive System	*ATP6V1E1, CFAP46, CLPTM1L, CPLX1, P2RX5, RPH3AL, STAG1, TUBB3*	8	9056	.60
Muscoskeletal System	*ATP6V1E1, GRIN1, P2RX5, PLEKHG4B, PTPRN2, STAG1, TUBB3*	7	7911	.59
				
Diseases				
Schizophrenia	*DLGAP2, EHMT1, CPLX1, GRIN1*	4	464	4.25
Autosomal Dominant Non-Syndromic Intellectual Disability	*GRIN1, PRICKLE2, EHMT1*	3	174	3.95
Benign Epilepsy with Centrotemporal Spikes	*GRIN1, PRICKLE2*	2	148	3.18
Neurodevelopment Disorder with or Without Hyperkinetic Movements and Seizures, Autosomal Dominant	*GRIN1*	1	1	2.83
Cutis Laxa, Autosomal revessive Type Lic	*ATP6V1E1*	1	1	2.83
				
Pathways				
MTHFR Deficiency	*GRIN1, EHMT1*	2	21	12.53
Activation of NMDA Receptor and Postsynaptic Events	*GRIN1, TUBB3*	2	93	8.29
Neuroscience	*GRIN1, TUBB3, CPLX1*	3	341	8.18
Transmission Across Chemical Synapses	*GRIN1, TUBB3, CPLX1*	3	410	7.44
S1P4 Pathway	*S1PR5*	1	8	7.12
				
Go Terms				
Positive Regulation of Calcium Ion transport Into Cytosol	*GRIN1, P2RX5*	2	13	13.90
Postsynapse	*P2RX5, CPLX1, CYP46A1*	3	137	11.95
Regulation of Exocytosis	*CPLX1, RPH3AL*	2	37	10.90
Synapse	*DLGAP2, GRIN1, CYP46A1, CPLX1, PTPRN2*	5	823	10.45
Terminal Bouton	*GRIN1, CPLX1*	2	46	10.28
				
HPO Phenotypes				
Abnormal Hippocampus Morphology	*GRIN1, CPLX1, TUBB3, PRICKLE2*	4	72	20.84
Abnormal Morphology of the Limbic System	*GRIN1, CPLX1, TUBB3, PRICKLE2*	4	73	20.76
Stereotypy	*EHMT1, GRIN1, CPLX1, TUBB3, PRICKLE2*	5	263	18.21
Ventriculomegaly	*EHMT1, GRIN1, ATP6V1E1, CPLX1, TUBB3, PRICKLE2*	6	679	15.36
Feeding Difficulties	*EHMT1, GRIN1, STAG1, ATP6V1E1, CPLX1, TUBB3, PRICKE2*	7	1080	14.94

*HPO*, human phenotype ontology; *DMPs*, differently methylated positions; *DMRs*, differently methylation regions; *MWAS*, methylome-wide association study.

## Discussion

We conducted a genome-wide methylation study in peripheral blood cells to investigate the association between DNA methylation and antipsychotic response. This study identified 17 DMPs and 21 DMRs, despite the use of peripheral blood cells, most of which were linked to genes involved in synaptic function and neurotransmitters. The combination strategy of position- and region-based analysis yielded notable findings that overlap a gene set enriched for biological processes highly relevant to antipsychotic response etiology.

Our results support the hypothesis that the mechanism of action of antipsychotic drugs might be mediated mainly by the neurotransmitter systems (Brandl et al., 2014). Additionally, the majority of the genes discovered were related to synaptic function. One of our top DMP findings was in the *CYP46A1* gene (Table 1), which encodes a member of the cytochrome P450 superfamily of enzymes. CYP46A1 may promote steady-state cholesterol in neurons, activate the mevalonate pathway, and coordinate the synthesis of new cholesterol and non-sterol involved in synaptic activity and learning (Mast et al., 2003; Mast et al., 2017). In addition, studies have shown that *CYP46A1* may be controlled epigenetically (Shafaati et al., 2009; Milagre et al., 2010; Nunes et al., 2010) and by specific transcription factors at the basal expression level (Milagre et al., 2008; Milagre et al., 2012). Taken together, our findings indicated that aberrant DNA methylation of *CYP46A1* may have an impact on its expression, thereby contributing to antipsychotic response etiology. We also identified *STAG1* as a gene potentially involved in antipsychotic response etiology. Singh et al. (Singh et al., 2022) demonstrated that several common and rare variants showed a convergence in *STAG1*that was significantly associated with schizophrenia. In addition to DMP-overlapping genes, we also found that two DMPs were located at the chromosome 16p11.2 region. Accumulating evidence suggests that the chromosome 16p11.2 region is implicated in schizophrenia (McCarthy et al., 2009; Chang et al., 2017; Marshall et al., 2017). Importantly, several studies reported regulatory effects of 16p11.2 copy number variations on dendritic spine development and synaptic function (Escamilla et al., 2017; Yadav et al., 2017; Richter et al., 2019). In addition, *RNF40* located at the 16p11.2 region was associated with the risperidone response in children with autism spectrum disorders (Lit et al., 2012).

Comparing these results within the DMP-antipsychotic response literature is difficult because a majority of the studies used candidate gene approaches. The only genome-wide study used the Agilent Human DNA Methylation Microarray platform to compare the methylation profiles of peripheral blood in the same subjects before and after antipsychotic treatment to controls (Rukova et al., 2014). No methylation alterations were significantly linked with treatment response, likely due to a small sample size (*n* = 20 blood samples) (Rukova et al., 2014). Small sample sizes are common in pharmacoepigenetic studies because of difficulties of sample recruitment and technology costs, but DMR analysis can mitigate power issues caused by small sample sizes by lessening the burden of multiple tests. We discovered that regional DNA methylation changes are more likely to contribute to the antipsychotic response in the DMR analysis. Furthermore, some DMR-overlapping genes identified were related to synaptic function. The most significant DMR corresponded to the *PTPRN2*, which is required for normal accumulation of the neurotransmitters norepinephrine, dopamine and serotonin in the brain (Rukova et al., 2014). EHMT1 is a histone methyltransferase that can mark the genomic region packaged with methylated histones (such as histone H3) for transcriptional repression. Defects in *EHMT1* are a cause of Kleefstra Syndrome, a genetic disorder linked with schizophrenia (Akbarian and Huang, 2009). In addition, *Drosophila EHMT1* mutants exhibited decreased dendrite branching of sensory neurons and impaired short and long-term memory that were reversed by restoring EHMT expression (Kramer et al., 2011). We also identified another target gene, *GRIN1*, which encodes a member of the glutamate receptor channel superfamily. GRIN1 is involved in synaptic plasticity, which is thought to underpin memory and learning. The glutamate receptor subunit GRIN2A was recently found to be associated with schizophrenia in the largest GWAS of schizophrenia so far (Trubetskoy et al., 2022). DLGAP2 is a membrane-associated protein that may play a role in synapse organization and signaling in neuronal cells. Li et al. found that DNA methylation of *DLGAP2* was associated with tardive dyskinesia caused by antipsychotic medications (Li et al., 2018). The results of enrichment analysis of annotated genes within DMRs were consistent with that of the DMP-related gene analysis, indicating that a network of neuronal-functioning genes contributes to the antipsychotic response.

Our gene set analysis revealed multiple lines of evidence linking differentially methylated genes to the antipsychotic response. Additionally, various biological processes, such as MTHFR deficiency, were also enriched for genes with aberrant methylation, warranting further investigation. The thoroughly characterized databases have provided vital data for constructing highly interconnected PPI networks that could reveal the underlying biological processes. Therefore, we investigated the interaction between differentially methylated genes and various known antipsychotic response risk genes. We found that the top susceptibility genes of antipsychotic response identified by GWAS (Yu H. et al., 2018) encoded a densely interconnected PPI network. Intriguingly, the DMP- and DMR-overlapping genes were also involved in this network, indicating their potential involvement in the common molecular network modulating the mechanism of antipsychotic response.

A crucial consideration for interpreting DNA methylation results is the tissue source (Michels et al., 2013; Lappalainen and Greally, 2017). It is suggested that CpGs from specific genomic regions had a statistically significant correlation between brain and peripheral blood samples (Walton et al., 2016). Notably, we found significantly enriched GO terms and pathways for the DMP- and DMR-overlapping genes, suggesting that the development of certain brain regions may be reflected in the DNA methylome of blood samples. According to the findings of gene set analysis, certain epigenetic markers in brain tissue can be mirrored by the corresponding sites in peripheral blood samples.

Validation analysis is critical for DNA methylation association studies to confirm preliminary findings. While no mass spectrometry-based bisulfite sequencing was completed, the literature was searched extensively for associations between antipsychotic response and biological signatures. As previously mentioned, a DMP-overlapping gene (*STAG1*) identified in this study was also found in the largest exome sequencing study of schizophrenia thus far (Rukova et al., 2014); a DMR-overlapping gene (*GRIN1*) in this study and *GRIN2A* identified in the largest GWAS of schizophrenia thus far (Trubetskoy et al., 2022) all belong to the glutamate receptor subunit. The findings from a previous pharmacogenomic study (Yu H. et al., 2018) and MWAS (Li et al., 2021) in schizophrenia implicated biological processes associated with synaptic functions, neurotransmitter receptors, and schizophrenia susceptibility. Similarly, this study identified DMP- and DMR-overlapping genes integral to synaptic vesicle exocytosis, presynapse, excitatory postsynaptic potential, regulation of neurotransmitter secretion, neurotransmitter transmembrane transporter activity (e.g., *GRIN1, TUBB3, CPLX1*, *CYP46A1*) and risk for schizophrenia (e.g., *DLGAP2*, *EHMT1*, *CPLX1*, *GRIN1*). While these similarities do not provide direct replication, they do lend credence to the DNA methylation findings.

While this study identified interesting genes and pathways, there are limitations that are worth noting. First, the sample size was modest, which may limit the statistical power to conduct robust single-site analysis. However, several pharmacoepigenomic studies have identified a series of reliable findings with small sample sizes (Maltby et al., 2015; Maltby et al., 2018; Henderson-Smith et al., 2019). Second, some environmental factors known to influence epigenetic modifications (such as smoking, stress, and circadian rhythm) were not controlled in the analysis. Third, because not all probes have the potential to form regions, regional analysis is inherently limited (Ong and Holbrook, 2014). Finally, this study was limited in its evaluation of functional importance.

## Conclusion

In summary, we discovered a set of DMPs and DMRs that were linked to antipsychotic response. To our knowledge, the present study is the first MWAS of the risperidone response in patients with schizophrenia using a technology that provides good coverage of methylation sites across the genome. Our findings show how DNA methylation research can offer fresh perspectives to increase our understanding of antipsychotic response and produce biomarkers with the potential to enhance clinical efficacy.

## Data Availability

The original contributions presented in the study are included in the article/Supplementary Materials, further inquiries can be directed to the corresponding authors.
