# Supplementary material for: Methylome-wide association study of different responses to risperidone in schizophrenia
Source: Front Pharmacol. 2022 Dec 22;13:1078464. doi: 10.3389/fphar.2022.1078464 (PMC9815458; doi:10.3389/fphar.2022.1078464)
Supplement: Supplementary file 1 [file Table4.DOCX]

Supplementary Figure S1. Gene expression pattern of the five DMP-overlapping genes (*NME9*, *CPLX1*, *CYP46A1*, *PTPRN2*, *S1PR5*) in human brain in the Human protein atlas database.


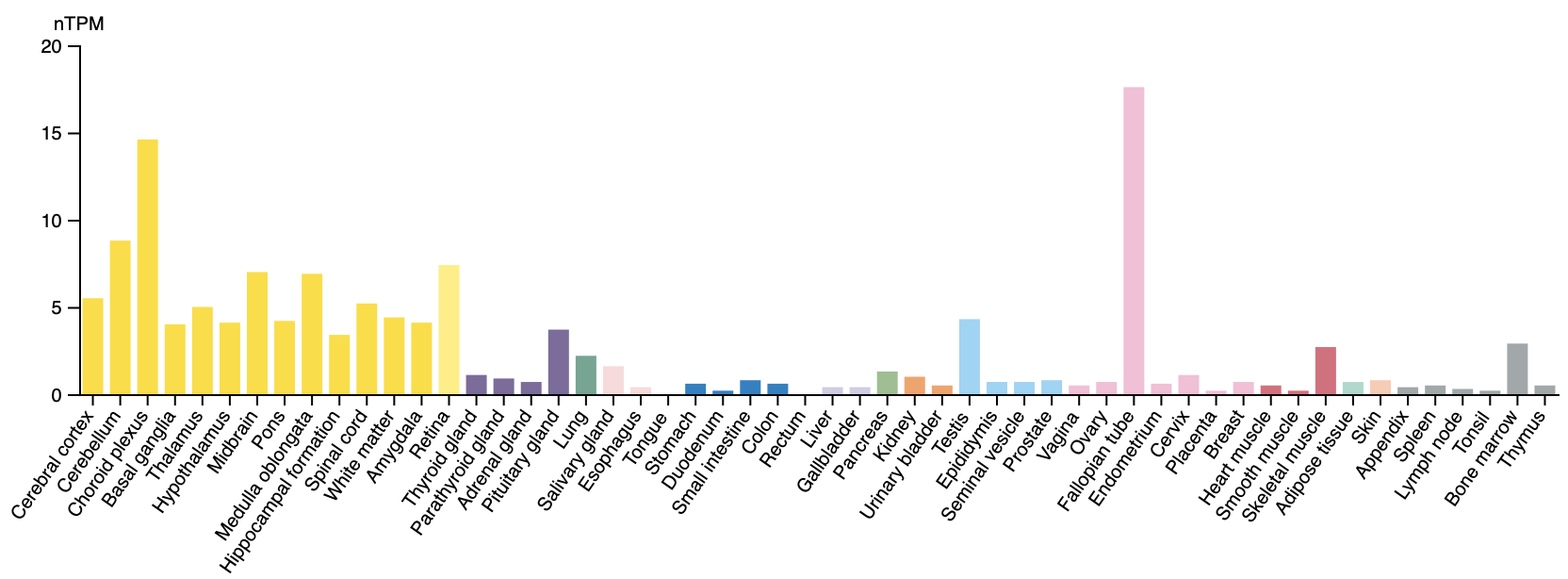


*NME9*


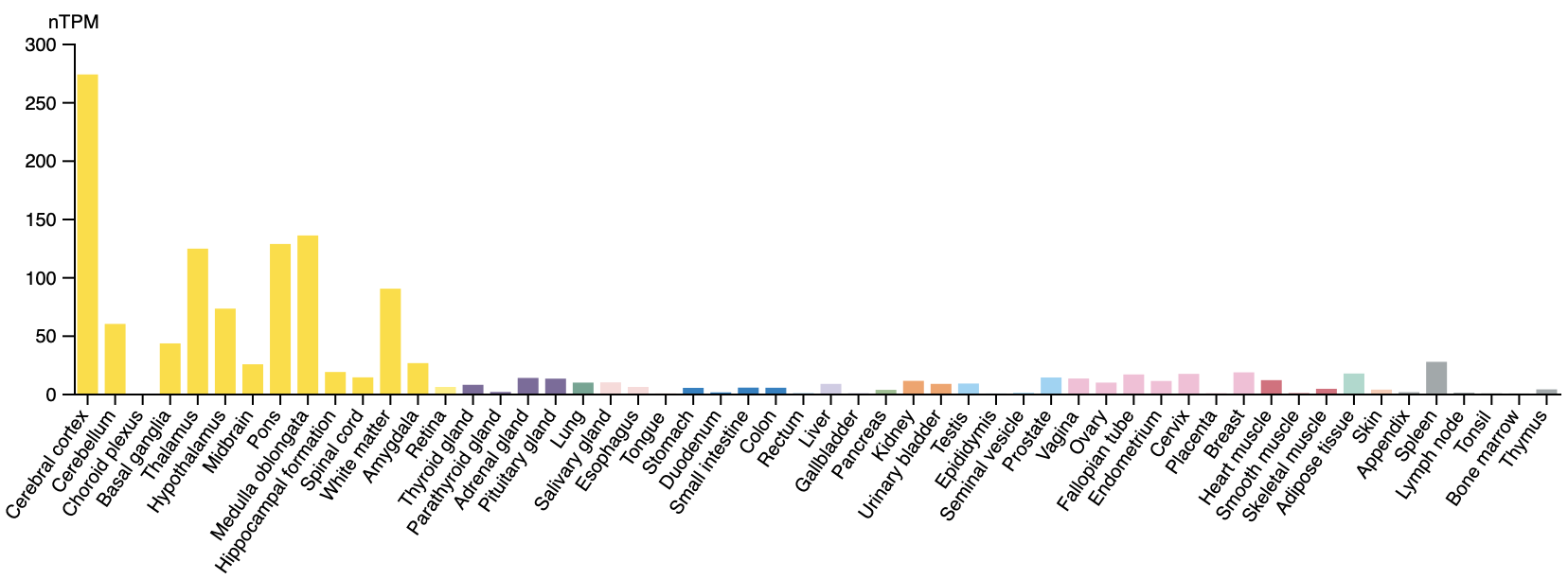


*CPLX1*


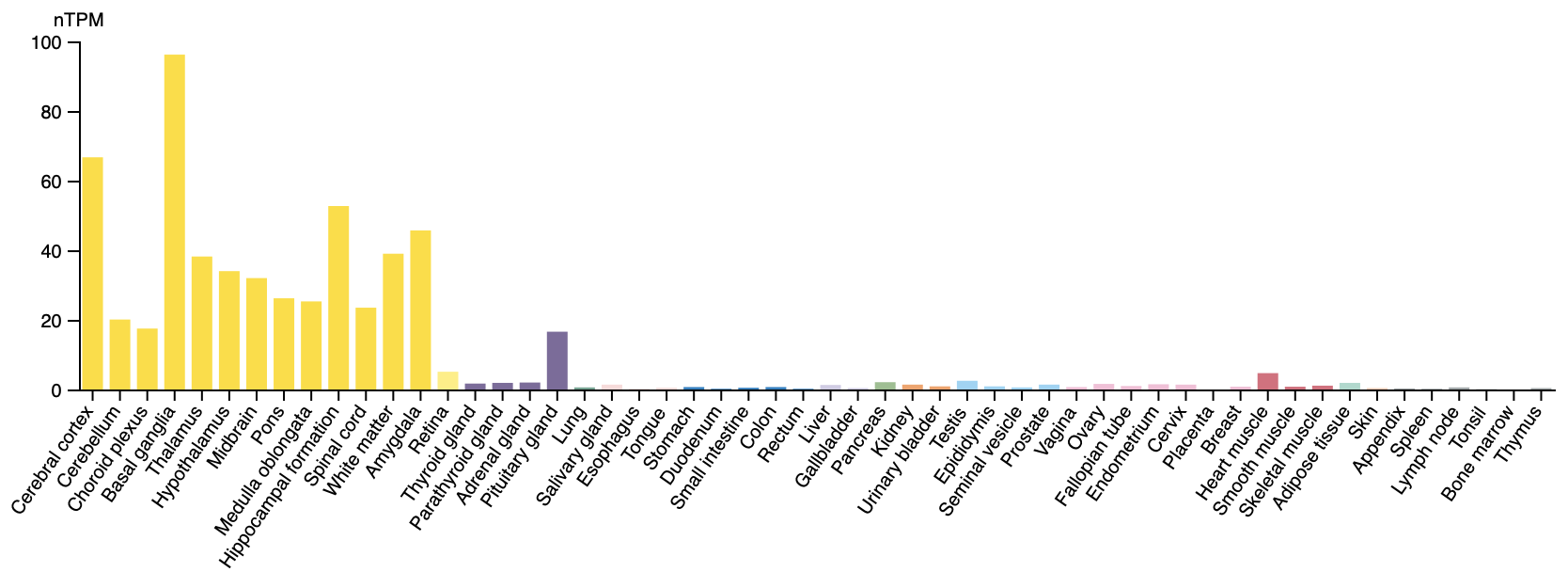


*CYP46A1*


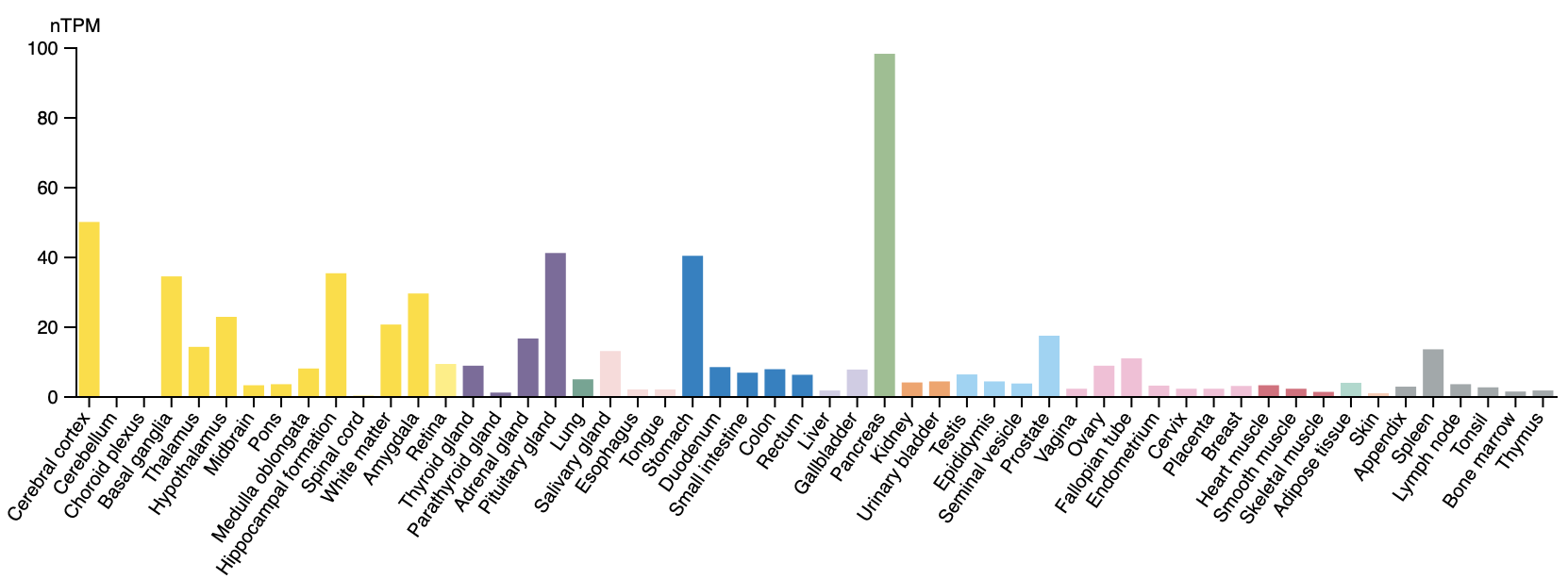


*PTPRN2*


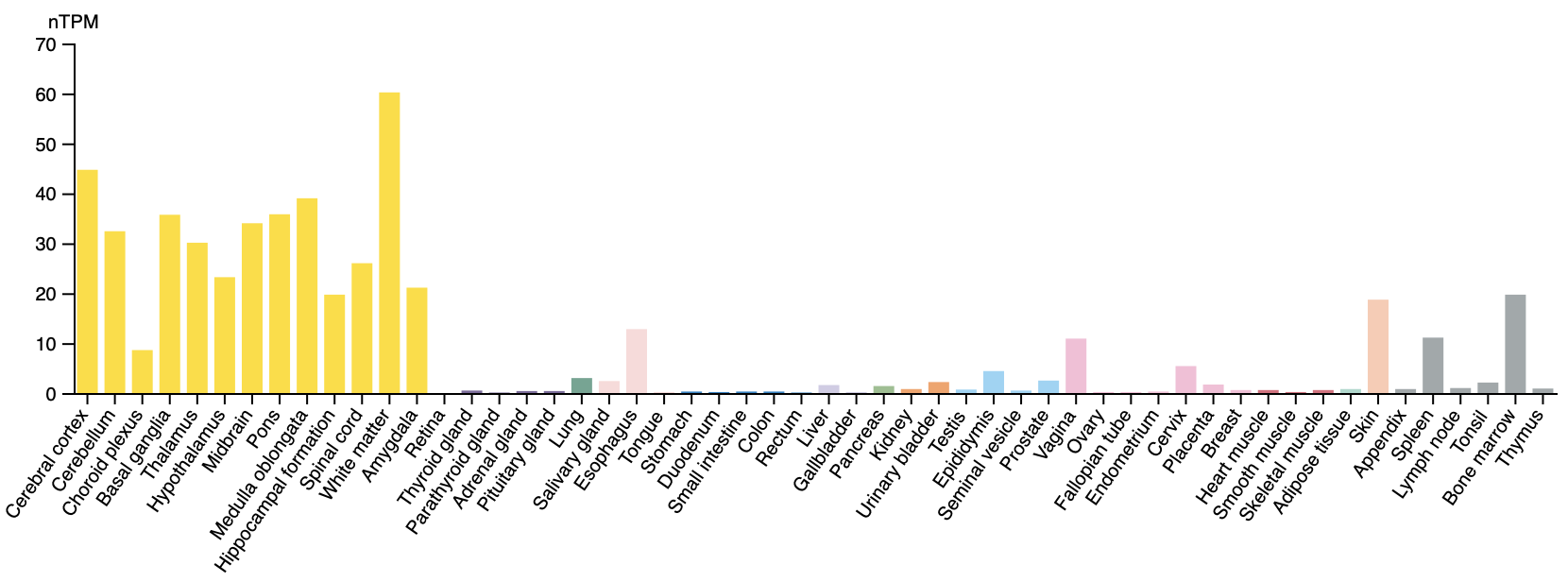


*S1PR5*

Supplementary Figure S2. Gene expression pattern of the six DMR-overlapping genes (*ATP9B*, *CFAP46*, *GRIN1*, *PLEKHG4B*, *PTPRN2*, *TUBB3*) in human brain in the Human protein atlas database.

*ATP9B*


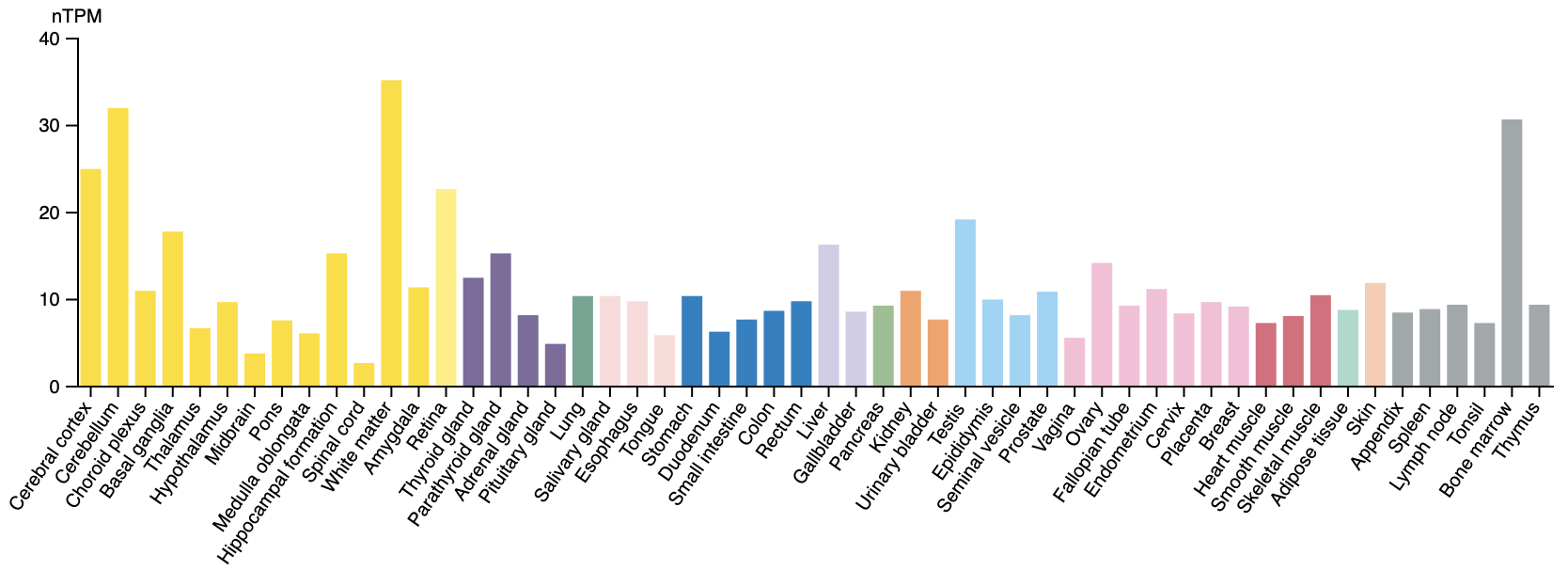


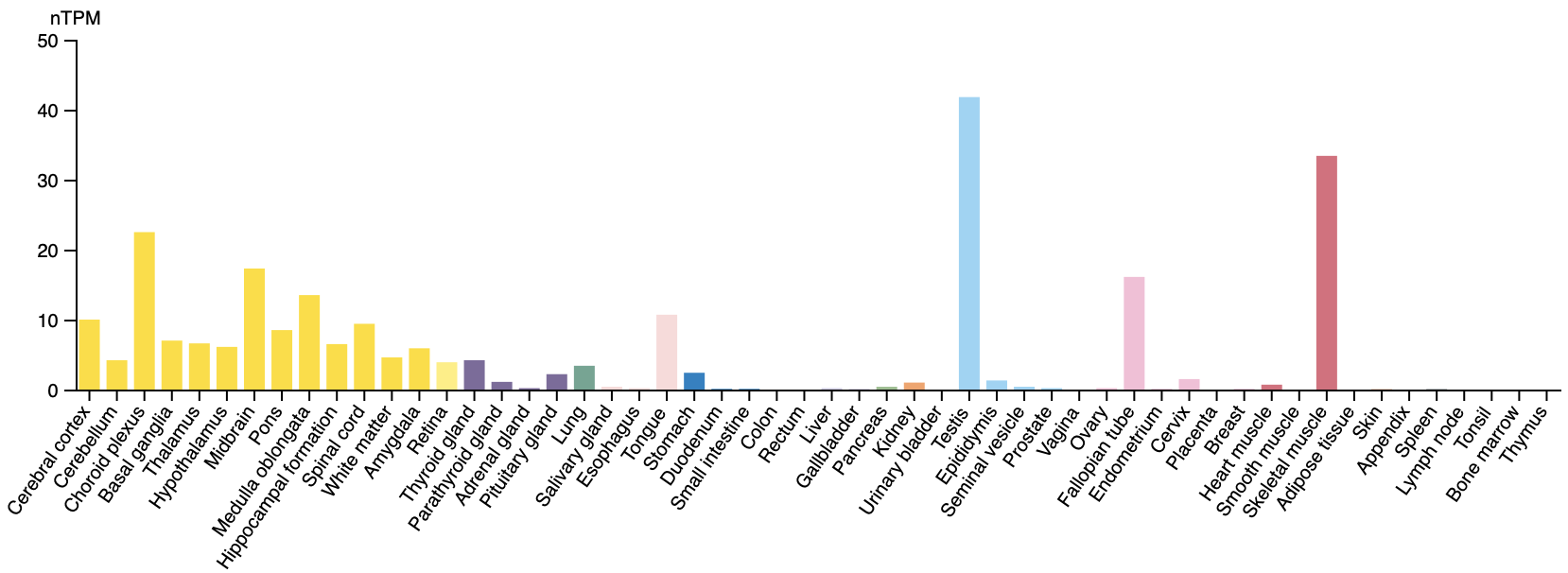


*CFAP46*


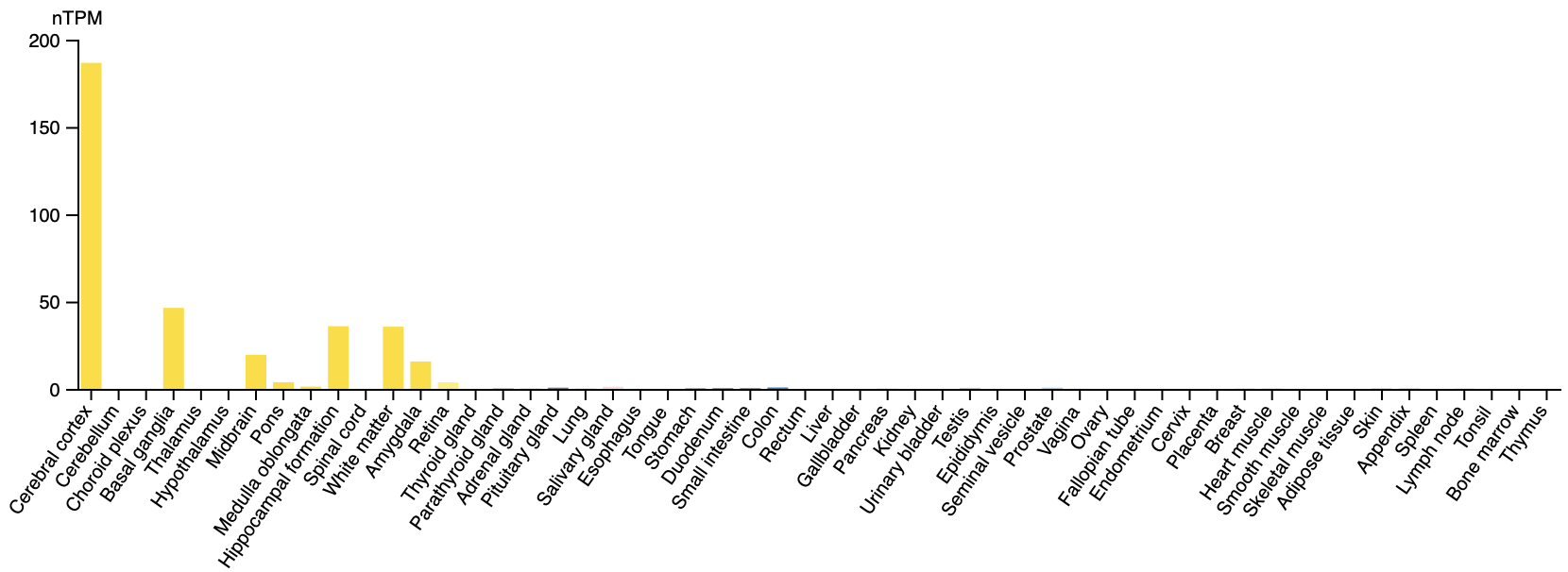


*GRIN1*


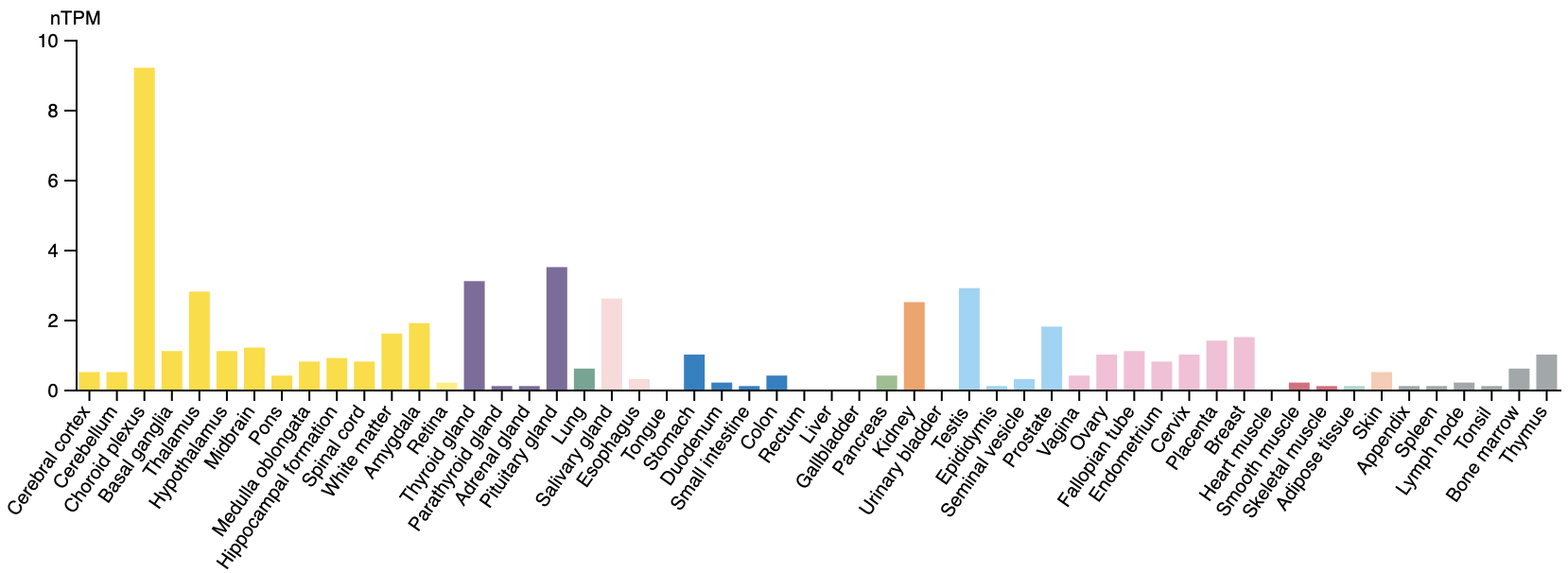


*PLEKHG4B*


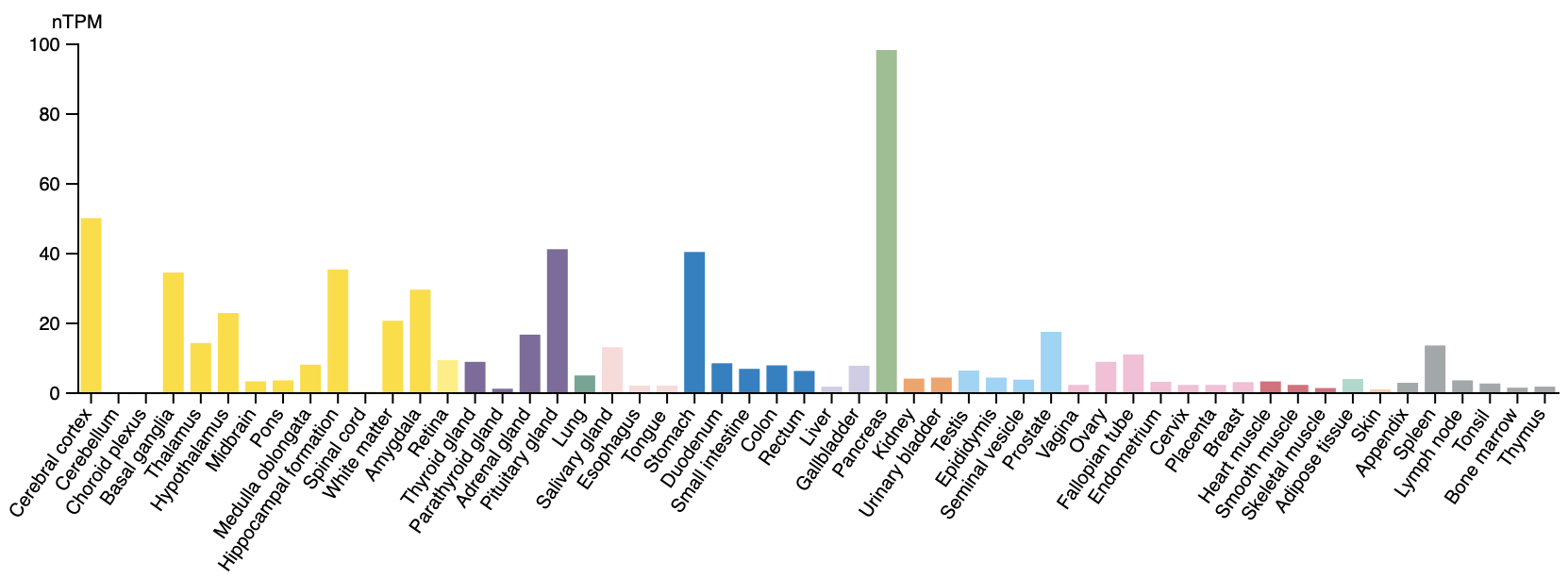


*PTPRN2*


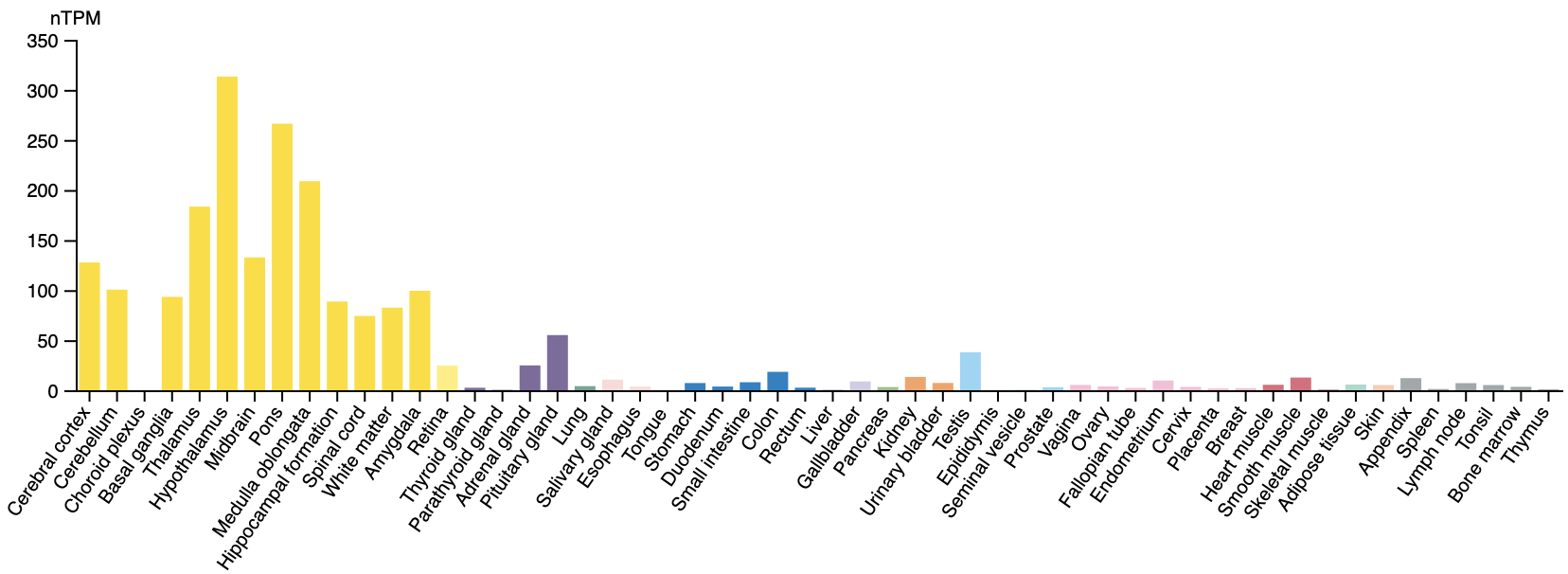


*TUBB3*

Supplementary Figure S3. Protein-protein interaction networks constructed by DMP-overlapping genes, DMR-overlapping genes, and candidate genes from published studies. (A) Red circle represents the DMP-overlapping genes; Yellow circle represents the DMR-overlapping genes; Grey circle represents other genes that interact with DMP- and DMR-overlapping genes. (B) Red circle represents the DMP-overlapping genes; Yellow circle represents the DMR-overlapping genes; Grey circle represents candidate genes. The sizes of circle represent extents of protein-protein association.


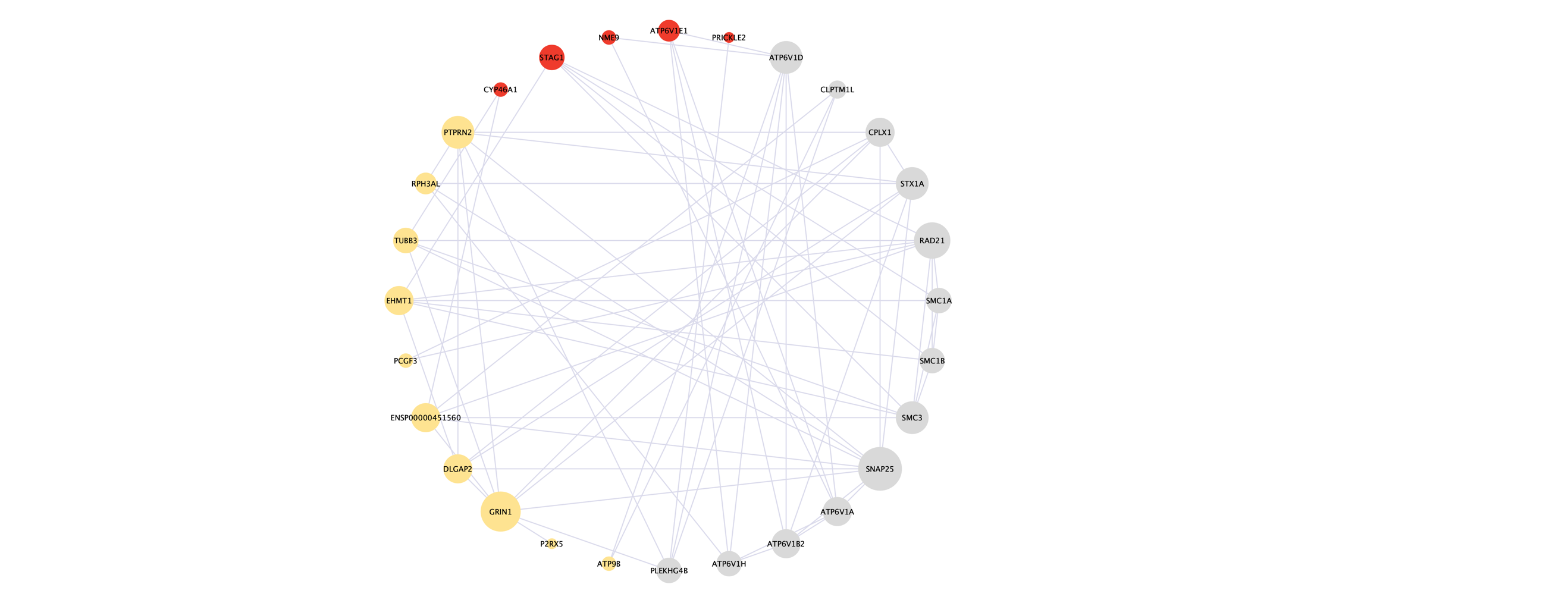

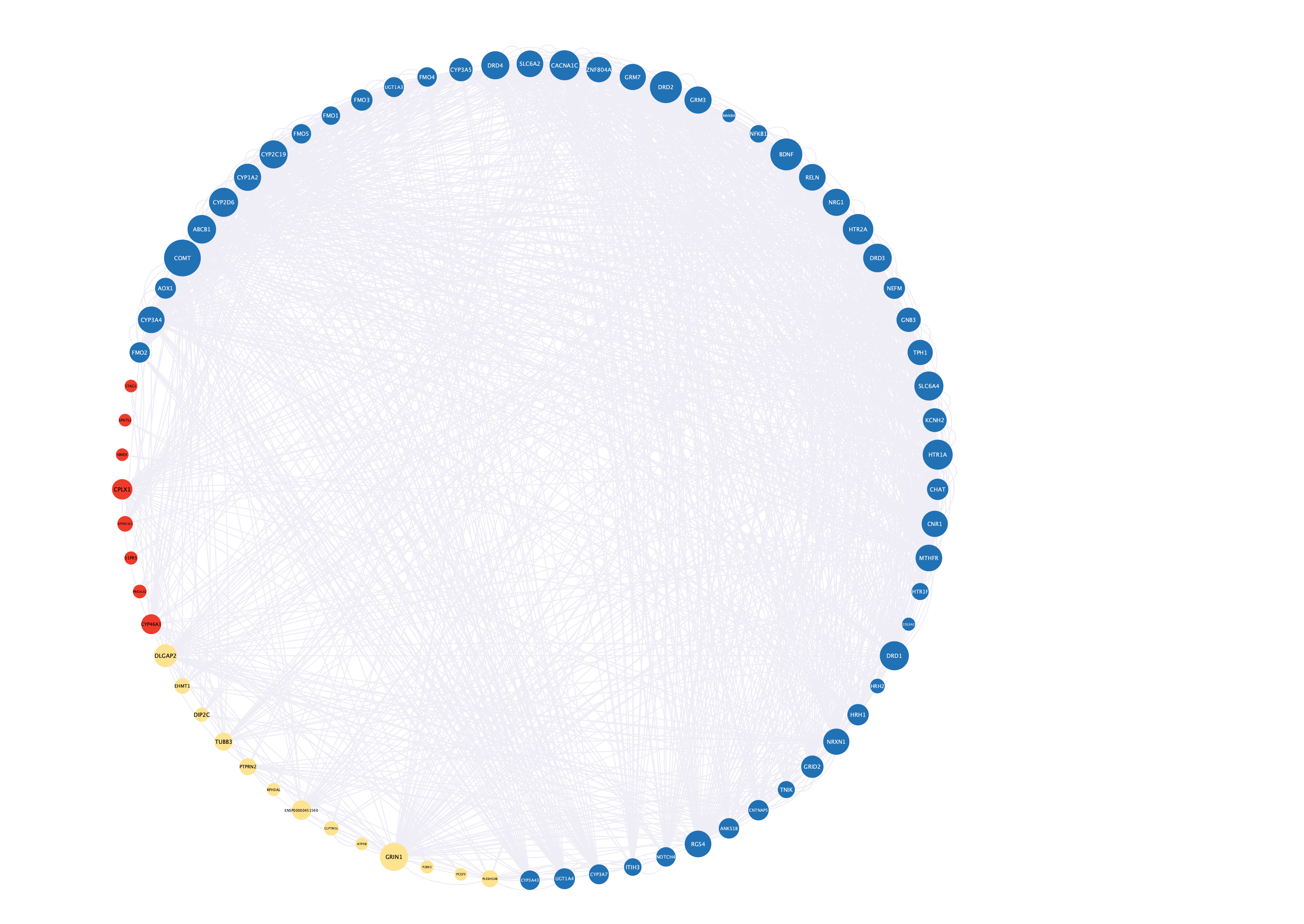


**B**

**A**
